# Endocrine autoimmunity in Turner syndrome

**DOI:** 10.1186/1824-7288-39-79

**Published:** 2013-12-20

**Authors:** Armando Grossi, Antonino Crinò, Rosa Luciano, Antonietta Lombardo, Marco Cappa, Alessandra Fierabracci

**Affiliations:** 1 Division of Endocrinology, Bambino Gesù Children’s Hospital IRCCS, Rome, Italy; 2 Research Laboratories, Bambino Gesù Children’s Hospital IRCCS, Rome, Italy; 3 Cytogenetics and Molecular Genetics Unit, Bambino Gesù Children’s Hospital IRCCS, Rome, Italy; 4 Autoimmunity Laboratory, Immunology Area, Bambino Gesù Children’s Hospital IRCCS, Piazza S. Onofrio, 4-00165 Rome, Italy

**Keywords:** Turner syndrome, Autoimmunity, Autoantibodies, Thyroid disease, Karyotype analysis

## Abstract

**Background:**

Turner syndrome is caused by numeric and structural abnormalities of the X chromosome. An increased frequency of autoimmunity as well as an elevated incidence of autoantibodies was observed in Turner patients. The aim of this study was to conduct a retrospective analysis of the incidence of autoimmunity in 66 Italian patients affected by Turner syndrome.

**Methods:**

Sixty-six unselected and consecutive Italian Turner patients were recruited. The association between age, karyotype and the presence of clinical/pre-clinical autoimmune disorders and of autoantibodies was examined.

**Results:**

Out of the 66 Turner patients, 26 had thyroid autoimmune disorders (39.4%), 14 patients had Hashimoto’s thyroiditis with clinical or subclinical hypothyroidism (21.2%) and 12 patients had circulating anti-thyroid antibodies, echographic pattern of diffuse hypoechogenicity and normal thyroid hormone levels (18.2%). None were affected by Graves’ disease. We analyzed the overall incidence of thyroid autoimmunity within the 3 different age groups 0–9.9, 10–19.9 and 20–29.9 years. No statistically significant difference was observed in the incidence of thyroid autoimmunity within the age-groups (χ^2^-test *p* > 0.05).

Out of the 66 patients, 31 patients had the 45,X karyotype; within this first group 14 out of 31 patients were affected by autoimmune thyroid disease. A second group of 29 patients included 19 patients with mosaicism, 5 patients with deletions and 5 patients with ring chromosome; out of these 29 patients 7 were affected by autoimmune thyroid disease. A third group included 6 patients with X isochromosome; 5 out of 6 were affected by autoimmune thyroid disease. A statistically significant difference in the frequency of thyroid autoimmunity within the different karyotype groups was observed (χ^2^-test *p* = 0.0173).

When comparing the X isochromosome group with the pooled group of other karyotypes, of note, the frequency of thyroid autoimmunity was statistically higher in the X isochromosome group (Fisher exact test *p* = 0.0315).

**Conclusions:**

Our data confirm a high frequency of thyroid autoimmunity in Italian Turner patients. Patients with X isochromosome are more prone to develop thyroid autoimmunity. Further, an early assay of autoantibodies and monitoring thyroid hormones is fundamental for detecting hypothyroidism earlier and start adequate replacement therapy.

## Background

Turner syndrome (TS) is a condition caused by numeric and/or structural abnormalities of the X chromosome [1]. Short stature and gonadal dysgenesis are the most frequent clinical features [2]. With a prevalence of 1:2,500 to 1:3,000 among live-born girls [3,4] and a 45, X karyotype found in approximately 10% of spontaneous aborptions (reviewed in [2,5]), TS is one of the most common chromosomal abnormality. Several studies report an increased frequency of autoimmunity in TS patients [3,6,7] possibly due to a complex interplay of genetic and environmental factors [6,8]. Haploinsufficiency of genes on the X chromosome could be responsible for lack of self-protein exposure in the thymus and escape of autoreactive T cells, thus predisposing to autoimmunity [6]. It has even been hypothesized that, since autoimmune disorders are frequent in relatives of families of TS patients, abnormal gametogenesis and non-dysjunctional events are due to abnormal autoimmune responsiveness [1,2,6].

Among autoimmune disorders associated with TS, Hashimoto’s thyroiditis has been estimated to affect around 50% of TS patients [2,9]. Other most commonly associated autoimmune disorders are: celiac disease (CD) [3,4], ulcerative colitis, Crohn’s disease, psoriasis, idiopathic thrombocytopenic purpura, vitiligo and juvenile rheumatoid arthritis [6]. In a recent investigation by Bakalov et al. [10], findings show that lymphocytic thyroiditis increases greatly in both, women with TS and women with karyotypically normal primary ovarian insufficiency (POI), suggesting that factors associated with ovarian insufficiency per se are responsible for this autoimmune condition. In addition the absence of a normal second X chromosome may contribute to increase the risk of autoimmunity in TS patients.

Thyroid autoimmune diseases are characterized by abnormal lymphocytic activation, directed against self-antigens, i.e. thyroglobulin (Tg) and thyroperoxidase (TPO) [11]. They encompasses Hashimoto’s thyroiditis (HT), a predominantly T cell mediated disease and Graves’ disease, characterized by a primarily humoral response and the presence of anti-thyroid stimulating hormone (TSH) receptor antibodies [11].

Although a higher prevalence of Hashimoto’s thyroiditis in TS patients was found in several studies, different percentages for the presence of circulating thyroid autoantibodies (Abs) and clinical diseases occur in various cohorts of patients investigated [12-15]. Moreover, in thyroid autoimmune disorders, a low prevalence of Graves’ disease was reported [12,16,17].

Interestingly, regarding the putative influence of karyotype on clinical features, some studies reported an association between autoimmune thyroiditis and the X isochromosome karyotype [12,17-21]; however, the small number of patients investigated limits its significance. This hypothesis was formulated on the basis that X isochromosome abnormality is more frequent in Hashimoto’s thyroiditis [12].

Contrary to studies that confirm a prevalent association between TS and thyroid autoimmunity, data from a Danish TS registry shows that when compared to Danish women in general, TS subjects had a 4-fold increased risk of developing male-predominant autoimmune diseases such as insulin dependent diabetes mellitus (Type 1 diabetes, T1D) [7]. Amyotrophic lateral sclerosis, ankylosing spondylitis, reactive arthritis and Dupuytren’s contracture are other male-predominant autoimmune diseases that could affect TS patients. This phenomenon indicates that while there is compensation by the normally functioning copy on the other X chromosome in women with a normal karyotype, a harmful allele will occur in TS females and males due to X monosomy [7]. To complete the spectrum of autoimmune abnormalities in TS, autoimmune polyendocrinopathy-candidiasis-ectodermal dystrophy syndrome (APECED) was also reported in a patient with premature ovarian failure (POF) and primary amenorrhea [22].

We conducted a retrospective analysis on the incidence of autoimmune manifestations in 66 Italian unselected and consecutive TS patients, recruited from the Bambino Gesù Children’s Hospital in Rome. The results were related to the age of the patients and their karyotype and will be discussed by comparing them with those reported in other studies.

## Methods

### Subjects

Sixty-six TS patients, aged 1–29.8 years, were recruited from the Bambino Gesù Children’s Hospital in Rome. All patients underwent chromosome analysis, which showed the presence of these karyotypes: 45,X (n = 31, 47%); mosaic (n = 19, 28.8%), with deletions (n = 5, 7.6%) and ring chromosomes (n = 5, 7.6%), and with isochromosomes (n = 6, 9.1%). Among the 31 patients with karyotype 45,X, SRY material was detected by FISH analysis in 2 patients. For one patient, as already reported in another study [1], standard karyotype analysis showed mosaicism for X monosomy and a complex rearrangement involving chromosome 2 and chromosome 10 (partial monosomy 2q and trisomy 10p). Karyotype was interpreted according to the International System for Human Cytogenetic Nomenclature (ISCN 2013).

Thyroid function was assessed in all patients at first presentation and at every follow-up visit. The definition of subclinical hypothyroidism was based on TSH levels >5.5 to 10 mcUI/ml [23] with normal FT_3_ (free triiodothyronine) and FT_4_ (free thyroxine) levels. The diagnosis of overt hypothyroidism was made when TSH values were higher than 5mcUI/ml and FT_3_ (free triiodothyronine) and FT_4_ (free thyroxine) levels were below the normal range. During the clinical follow-up patients exhibiting subclinical/overt hypothyroidism or with positive TPO and/or Tg antibodies underwent thyroid ultrasound. Diffuse low echogenicity was considered an indicator of thyroid autoimmune disease [24].

### Ethical issues

Informed consent was obtained from all TS patients or their parents if they were younger than 18 years. The investigation was approved by the Ethics Committee of the Hospital.

### Detection of autoantibodies

Sera of each patient were assessed for autoantibodies in parallel to thyroid function at first presentation and at every follow-up visit. Patients’ sera were tested for Abs to Tg and TPO by chemiluminescence method; parietal cells, adrenal cortex and islet cell by indirect immunofluorescence; steroid 21-hydroxylase (21-OH), glutamic acid decarboxylase (isoform 65) (GADA), protein tyrosine phosphatase IA2 (insulinoma-associated antigen 2) and insulin by RIA. Total IgA, IgA anti-gliadin and anti-transglutaminase were also measured for celiac screening [25].

### Statistical analysis

Qualitative variables were described as numbers and percentages. A χ^2^-test was used to compare frequency of qualitative variables among the different groups. A Fisher’s exact test was computed for 2x2 tables. Results were analyzed using the GraphPad Prism software version number 5 (San Diego, California, USA). A result with *p* < 0.05 was considered statistically significant.

## Results

### Autoimmune manifestations in TS patients

Autoimmune thyroid disease (ATD) was the most common autoimmune disorder.

Out of the 66 TS patients, 26 had thyroid autoimmune disorders (39.4%) (Table 1), 14 patients had Hashimoto’s thyroiditis with clinical or subclinical hypothyroidism (21.2%) and 12 patients had circulating anti-thyroid Abs, echographic pattern of diffuse hypoechogenicity and normal thyroid hormone levels (18.2%). None were affected by Graves’ disease. In addition, one patient had already reported [1] that she developed celiac disease at the age of 3.5 years and was also affected by clinical T1D, Hashimoto’s thyroiditis and alopecia universalis. One patient had circulating Abs against antigens related to T1D but no clinical manifestations of overt disease. One patient, at the age of 9.3 years, had developed anti-transglutaminase Abs that indicate CD and another patient was affected by vitiligo associated with thyroid autoimmunity.

**Table 1:** TS patients with thyroid autoimmunity across age-groups

**Age range**	**Number (n) of patients**	**Percentage of patients with thyroid autoimmunity**
**0–9.9 years**	12	16.7% (n = 2)
**10–19.9 years**	38	47.4% (n = 18)
**20–29.9 years**	16	37.5% (n = 6)

No statically significant difference in the incidence of thyroid autoimmunity was observed in the age groups.

Since thyroid autoimmunity was the most frequent autoimmune disorder, we analyzed the overall incidence of thyroid autoimmunity within the 3 different age groups 0–9.9, 10–19.9 and 20–29.9 years. As shown in Table 1, no statistically significant difference was observed in the incidence of thyroid autoimmunity within the age-groups (χ^2^-test = *p* > 0.05).

Table 2 illustrates the number of TS patients with thyroid autoimmunity within the different karyotype groups. Out of the total group of 66 patients, 31 patients had the 45, X karyotype; within this first group 14 out of 31 patients were affected by ATD (45.2%; 21.2% of the total group of patients). A second group of 29 patients included 19 patients with mosaicism, 5 patients with deletions and 5 patients with ring chromosome; out of these 29 patients 7 were affected by ATD (24.1%; 10.6% of the total group of 66 patients). A third group included 6 patients with X isochromosome; 5 out of 6 were affected by ATD (83.3%; 7.6% of the total group of 66 patients) (Table 2). As shown in Table 2, there was a statistically significant difference in the frequency of thyroid autoimmunity within the different karyotype groups (χ^2^-test *p* = 0.0173).

**Table 2:** Distribution of number of patients with thyroid autoimmunity in the different karyotypes

**Karyotype**	**Number of patients within the karyotype group**	**Number of patients with thyroid autoimmunity within the karyotype group**^(*)^	**Percentage of patients with thyroid autoimmunity within the karyotype group**	**Percentage of patients with thyroid autoimmunity in respect to the total group of patients**
**45,X**	31	14	45.2%	21.2%
**Mosaicism and others**	29	7	24.1%	10.6%
**X isochromosome**	6	5^(**)^	83.3%	7.6%

Autoimmunity prevalence is calculated over the total number of patients per karyotype and over the total 66 TS patients in each karyotype group^(*)^. There was a statistically significant difference in the frequency of thyroid autoimmunity within the different karyotype groups (χ^2^-test *p* = 0.0173). When comparing the X isochromosome group with the pooled group of other karyotypes, of note, the frequency of thyroid autoimmunity was statistically higher in this group^(**)^.

Nevertheless, although the analysis is limited by the small number of TS patients with X isochromosome, when comparing this group with the pooled group of other karyotypes, of note, the frequency of thyroid autoimmunity was statistically higher in the X isochromosome group (Fisher exact test *p* = 0.0315) (Table 2).

Regarding the overall incidence of organ-specific autoimmunity in our patients (28 out of 66, 42.4%), by adding one patient with anti-transglutaminase Abs [age 9.1, karyotype 46,del(X)(p11)] and the patient with islet-related Abs (age 16.2, karyotype 45,X), again no statistically significant difference was observed within the different age groups (χ^2^ Test, *p* > 0.05).

Interestingly, among patients affected by Hashimoto’s thyroiditis with clinical or subclinical hypothyroidism, 3 out of 14 patients developed clinical disease and are currently under substitutive treatment with levothyroxine (LT4) (Table 3).

**Table 3:** Characteristics of TS patients affected by Hashimoto’s thyroiditis

**Patient**	**Age (years)**	**Karyotype**	**Diagnosis**
PF	9.4	45,X	HT-SH
FG	10.3	mos 45,X[6]/46,X,i(X)(q10)[94]	HT-SH
MF	14.4	45,X	HT-SH
BI	15.4	mos 45,X[93]/46,X,i(X)(q10)[7]	HT-SH
DF	16.3	46,X,del(X)(p22)	HT-SH
BF	16.4	mos 45,X,der(2)t(2;10)(q37;p13)[18]/46,XX,der(2)t(2;10)(q37;p13)[82]	HT-SH
DE	18.3	mos 45,X[68]/46,XX[32]	HT-SH
IA	18.5	mos 45,X[26],46,XX[74]	HT-SH
SI	20.3	45,X	HT-SH
LL	27.6	45,X	HT-SH
SS	27.8	mos 45,X[44]/46,X,r(X)(p22.3q28)[56]	HT-SH
PV	13.2	mos 45,X[25]/46,X,r(X)(p11.1q13.1)[75]	HT-H
FS	17.1	mos 45,X[94]/46,XX[6]	HT-H
TM	25.7	mos 45,X[40]/46,X,i(X)(q10)[60]	HT-H

HT = Hashimoto’s thyroiditis; H = hypothyroidism; SH = subclinical hypothyroidism.

The case report related to patient BF was published in Reference [1].

Table refers to the age, karyotype and presence of clinical or subclinical hypothyroidism in TS patients affected by Hashimoto’s thyroiditis.

## Discussion

An increased incidence of autoimmunity has been largely documented in TS [3,7] (Table 4). In particular, an increased frequency of thyroid abnormalities has been reported; in addition, in several studies this frequency varied greatly. This wide range of results could be due to selection bias, inclusion of pediatric versus adult patients and karyotype differences. Generally, thyroid Abs can be detected in a percentage of patients that varies between 3.9 and 87.5%, while for thyroid dysfunction the percentage varies between 4.3 and 40% [26-29] as assessed by clinical and laboratory parameters; in the majority of studies the incidence of thyroid autoimmune disorders exceeded 20% [7,9,17,18,30,31].

**Table 4:** The epidemiology of autoimmune thyroid disease in Turner syndrome

**Reference**	**n of Turner patients**	**Age range or mean**	**Autoimmune diseases**	**Population origin**	**% of subjects with thyroid autoimmune disease**	**% of subjects with thyroid autoimmune disease in IsoXq**	**% of patients with clinical/subclinical hypothyroidism**	**% of patients with Graves’ disease**
Bright *et al.* 1982 [26]	24	15 ± 5	Thyroiditis	USA	87.5	100	na	na
Germain *et al.* 1986 [23]	100	15 weeks- 19 years	Thyroiditis	USA	50	57	22	0
Gluck *et al.* 1992 [27]	77	5-14	Thyroiditis	Germany	3.9	na	na	na
de Kerdanet *et al.* 1994 [21]	67	5.5-34	Thyroiditis	France	26.9	68.8	23.9	na
Radetti *et al.* 1995 [30]	478	3.6-25.3	Thyroiditis	Italy	22.2	na	6.1	0.6
Chiovato *et al.* 1996 [12]	75	3-30	Thyroiditis	Italy	13.3	40	10.6	1.3
Medeiros *et al.* 2000 [13]	71	0-20	Thyroiditis	Brazil	23.9	na	15.5	0
Medeiros *et al.* 2009 [14]	17**/**80 (follow-up)	5.9-22.6	Thyroiditis	Brazil	59	33	82.4	5.8
Elsheikh *et al.* 2001 [18]	145	16-52	Thyroiditis	UK	41	83	15	0.7
El-Mansouri *et al.* 2005 [31]	91	25-65	Thyroiditis	Sweden	27.5	27.8	25	2
Livadas *et al.* 2005 [17]	84	0-19	Thyroiditis	Greece	60.7	71	24 21 with Abs	2.5
Bettendorf *et al.* 2006 [15]	120/327 (follow-up)	>16	Presence of thyroid and celiac disease Abs	Germany	36	5.8	57.1	0
Mc Carthy *et al.* 2008 [3]	100	7-17	Thyroiditis	USA	51	na	na	na
Fukuda *et al.* 2009 [36]	65	15-61	Thyroiditis	Japan	57	na	31	4.6
Mortensen *et al.* 2009 [28]	107	6-60	Thyroiditis, celiac disease	Denmark	45	39	15	1.9
Dias *et al .* 2010 [4]	56	0.8-52	Celiac disease	Brazil	na	na	na	na
Jөrgensen *et al.* 2010 [7]	798	na	Female predominant	Denmark	1.6	12.5 with	na	0.6
						Hashimoto’s		
			i.e. Hashimoto’s thyroiditis			thyroiditis		
			Male predominant i.e.T1D,					
			Ulcerative					
			Colitis					
			Dupuytren’s contracture,					
			Amyotrophic lateral					
			Sclerosis, reactive arthritis					
Gawlik *et al.* 2011 [35]	86	0-17.4	Thyroiditis, celiac disease, T1D, alopecia	Poland	36 with Abs 17.4 with Hashimoto’s thyroiditis	35.3 with Abs 17.6 with autoimmune thyroid disease	31.4	0
Bakalov *et al.* 2012 [10]	224	18-67	High prevalence of Hashimoto’s thyroiditis, inflammatory bowel disease and celiac disease	USA	37	58.3	na	0
Hamza *et al.* 2012 [20]	80	4.7-22.3	Thyroiditis	Egypt	35 anti-TPO 15 anti-Tg	57.9 anti-TPO 26.3 anti-Tg	6.3	1.3
Kucharska *et al.* 2013 [29]	54	11.9 ± 2.5	Thyroiditis	Poland	64.8 anti-TPO 24 anti-Tg 14.8 anti-NIS	na	20	0

N = number; na = not available; NIS = natrium/iodide symporter.

Table reports-a summary of the literature on the incidence of autoimmune thyroid disease in Turner patients in different population studies.

By analysing a group of 66 TS patients, including children, adolescents and young adults up to the age of 29.9 years, we observed an overall increased incidence of organ-specific autoimmunity. We need to point out that the main limitation of our study is its retrospective character and the limited number of patients included. However this allowed timely comparison with other relevant studies that were published in the field within the past twenty years and include similar numbers of patients (Table 4).

The observed incidence of thyroid autoimmunity in our study was 39.4%; 21.2% of patients were affected by clinical Hashimoto’s thyroiditis and 18.2% by preclinical autoimmune thyroiditis. This latter is characterised by the presence of circulating thyroid autoantibodies, normal thyroid hormone levels and/or echographic pattern of diffuse hypoechogenicity. These data clearly confirm an increased incidence of thyroid autoimmunity in TS patients from the estimated incidence of 4.9% of females in the Italian population as assessed by high antibody titer and hypoechogenic ultrasound in the Pescopagano survey [32-34]. There was no statistically significant difference in the frequency of autoimmunity among the different age groups.

In comparison with other relevant investigations, in a study by Chiovato et al. [12], conducted on 75 Italian TS patients, thyroid abnormalities and thyroid Abs occurred in 20% and in 40% of individuals respectively. The frequency of thyroid Abs increased with age from 15% in patients younger than 10 years to 30% in patients older than 20 years. The prevalence of autoimmune disease increased significantly after the age of 13 years, to reach its peak in the third decade of life [12].

Similarly in a recent report on the Polish TS population by Gawlik et al. [35], 31% of TS patients had circulating thyroid Abs at the median age of 14.1 years, while 17% of patients developed hypothyroidism as a consequence of Hashimoto’s thyroiditis. Autoantibodies appeared mainly after the age of 13 years, but from the beginning of the observation to the end of the follow-up, this frequency increased from 25.5 to 50%.

In a Japanese study by Fukuda et al. [36], 65 TS women (age 30 ± 9 years old, range 15–61) were retrospectively evaluated: more than half of adults had thyroid autoantibodies (57%). Again, the study found an age-related increase of thyroid Abs. The prevalence was 7% below 30 years of age and 14% above 30 years. An investigation conducted in Egypt in 2012 [20], showed that out of a cohort of 80 TS patients, 67.5% were seropositive for one or more Abs.

It is generally recognised that autoimmune thyroiditis has etiopathogenetic mechanisms that are similar to those of Graves’ disease. In keeping with previous studies [12,17,20], no cases of Graves’ disease were observed in our investigation. By contrast, Graves’ disease was observed in 2 out of 84 TS patients (2.5%) versus 0.5-1% in the general population in a study by Livadas et al. [17]. In the same study by Fukuda et al. mentioned above [36], out of 65 TS women, 3 had Graves’ disease and 31% was hypothyroid. Similarly, only one Graves’ disease patient was reported by Chiovato et al. [12] out of 75 TS patients investigated.

Although statistical significance was not achieved, according to several studies, the overall frequency of thyroid autoimmunity was higher in TS patients with X isochromosome i(X)(p10) karyotype [12,17-20].

In keeping with previous studies, in our investigation the group with X isochromosome had a number of patients affected by thyroid autoimmunity higher than in the other karyotype groups. It is noteworthy that in the investigation by Chiovato et al. [12], the only patient with Graves’ disease had the X isochromosome karyotype. In the study by Livadas et al. [17], 71% of patients with IsoXq had positive Abs and over 41 to 47% presented the other karyotipic abnormalities. In a study by Hamza et al. [20], there was no statistically significant association between karyotype 45,X and the overall prevalence of Abs. On the contrary, a significant association was confirmed between IsoXq and the increased prevalence of anti-TPO, anti-Tg Abs and GADA, whereas, this karyotype was not associated with anti-transglutaminase Abs.

We observed thyroid autoimmunity in one patient under 8 years of age (6.9 years old). Thus we recommend that thyroid evaluation and testing of related Abs in girls with TS should be carried out even before the age of 8 years. Some studies report evidence that there is no incidence of thyroid Abs and/or hypothyroidism before the age of 8 years [9,17,31]. In the study by Livadas et al. [17], thyroid dysfunction occurred more frequently in girls with TS, and especially with i(X)(q10). Thyroid dysfunction was manifested early, at the age of 8 years. In other studies, hypothyroidism was detected in girls before the age of 10 years, while de Kerdanet et al. [21], detected the disease in patients older than 10 years.

Finally, we observed that in our cohort of TS patients, the high incidence of autoimmune diseases that are normally characterised by male preponderance as observed in the Danish TS registry [7], was not present in our cohort of TS patients. In addition, one patient developed CD at the age of 3.5 years [1] and another presented anti-transglutaminase Abs at the age of 9.3 years. Although this positivity for CD screening (3%) is lower than that reported in other studies [10,34], its screening should be part of the routine follow up of girls affected by TS since the first years of life.

## Conclusions

Our data confirm a high frequency of thyroid autoimmunity in Italian Turner patients. Patients with X isochromosome are more prone to develop thyroid autoimmunity. Further, an early assay of autoantibodies and monitoring thyroid hormones is fundamental for detecting hypothyroidism precociously and start adequate replacement therapy.

## Abbreviations

Abs: Autoantibodies; APECED: Autoimmune polyendocrinopathy-candidiasis-ectodermal dystrophy syndrome; ATD: Autoimmune thyroid disease; CD: Celiac disease; FT3: Free triiodothyronine; FT4: Free thyroxine; FISH: Fluorescence in situ hybridization; GADA: Glutamic acid decarboxylase (isoform 65); H: Hypothyroidism; HT: Hashimoto’s thyroiditis; IA2: Insulinoma-associated antigen 2; IgA: Immunoglobulin A; ISCN: International system for human cytogenetic nomenclature; LT4: Levothyroxine; mcUI/ml: Micro internation units/milliliter; n: Number; na: Not available; neg: Negative; NIS: Sodium iodide symporter; TS: Turner syndrome; POF: Premature ovarian failure; POI: Primary ovarian insufficiency; RIA: Radioimmunoassay; SH: Subclinical hypothyroidism; SRY: Sex-determining region Y; Tg: Thyroglobulin; TPO: Thyroperoxidase; TSH: Thyroid stimulating hormone; T1D: Type 1 diabetes; 21-OH: 21-hydroxylase.

## Competing interests

The authors declare that they have no competing interests.

## Authors’ contributions

AG and AF designed the study, carried out the data elaboration, coordinated the study and drafted the manuscript. MC and AC revised the manuscript critically for important intellectual content. RL has been involved in the analysis and interpretation of data. AL conducted cytogenetic studies. All authors read and approved the manuscript.
